# HPB News

**DOI:** 10.1155/1990/43916

**Published:** 1990


					HPB Surgery, 1990, Vol. 2, pp. 225-226
Reprints available directly from the publisher
Photocopying permitted by license only
1990 Harwood Academic Publishers GmbH
Printed in the United Kingdom
HPB NEWS
The General Assembly of the Members of the Italian Chapter of the World
Association of H.P.B. Surgery was held in Bologna on December 16, 1989.
COMMUNICATIONS OF THE PRESIDENT
The President of the Italian Chapter, Giuseppe Gozzetti, informed the members of
his requests to the International Board in order to obtain a more consistent
involvement of Italian members in the activities of the Association and in the next
World Congress in London.
He then informed the audience about the progression of the multicentric study
on biliary tract cancer in Italy. The preliminary enquiry conducted in specialized
Centers, received unanimous consents.
Starting from 1990 the journal Chirurgia Epatobiliare founded by Prof.
Aureliano Puglionisi, will be the official journal of the Italian Chapter.
Finally, the President informed the audience that the next congress of the Italian
Chapter will be held in Taormina, on June 6-8, 1991. Chairman of the Congress
will be Prof. Gactano Romeo.
BOARD OF THE ITALIAN CHAPTER
In order to allow completion of programs under way, the President proposed to the
Assembly that the actual Board of the Italian Chapter be reconfirmed until 1993.
The candidatures and composition of the successive Board will be discussed in
Taormina, during the next Congress of the Italian Chapter.
The Assembly approved unanimously the President's proposals. The Board of
the Italian Chapter will thus remain as follows for the next four years.
President: G. Gozzetti
Vice President: D. D'Amico
General Secretary: P. Magistrelli
Treasurer: R. Masetti
Members: R. Cavaliero
L. Gennari
A. Picciocchi
A. Principe
G. Romco
M.L. Santangelo
A.M. Taschieri
225
226 HPB NEWS
THE FIRST ASIAN CONFERENCE OF HPB SURGERY
The First Asian Conference of HPB Surgery will take place at Central Plaza Hotel
in Bangkok, Thailand on January 10 to 12, 1991.
Specific topics to be covered are
1. Biliary Calculi including Hepatolithiasis
2. Biliary Infection
3. Hepatocellular Carcinoma
4. Acute Pancreatitis
5. Ideal techniques in HPB Surgery
6. Others Video and Plenary sessions
Deadline for abstract: September 30, 1990
Official language, English
Organizing Committee
R. Tsuchiya, Y. Saito, F. Hanyu, Advisors; T. Takada, Director; T. Yamakawa,
Co-director; T. Nakayama, K. Satake, Y. Kawarada, T. Nagakawa, K. Takasaki,
M. Ryu, Y. Nimura, Y. Ogura, Scientific advisors.
Additional information
contact T. Takada, M.D., F.A.C.S.
1st Department of Surgery,
Teikyo University School of Medicine,
2-11-1, Kaga, Itabashi-ku, Tokyo 173 Japan
Fax: 03-962-2128
ERRATUM
Letter to the Editor Vol.1, pp. 257-258
This letter, from J.D. Greig and J.R. Goldring, appeared with incomplete infor-
mation for those wishing to contact the authors.
The letter concerned the paper by Andersson and colleagues entitled "Haemo-
peritoneum after spontaneous rupture of liver tumor: results of surgical treat-
ment". HPB Surgery 1988:1:81-83.
Please note that any correspondence, or any requests for the letter should be
addressed to:
Mr. J.D. Greig
University Department of Surgery
Royal Infirmary of Edinburgh
Lauriston Place
Edinburgh EH3 9YW

				

